# Supplementary figures and images for: Cone photoreceptor ablation in microglia-deficient larval zebrafish retina elicits a regenerative response alongside a compensatory immune cell response
Source: PLoS One. 2026 Jul 23;21(7):e0344737. doi: 10.1371/journal.pone.0344737 (PMC13395315; doi:10.1371/journal.pone.0344737)

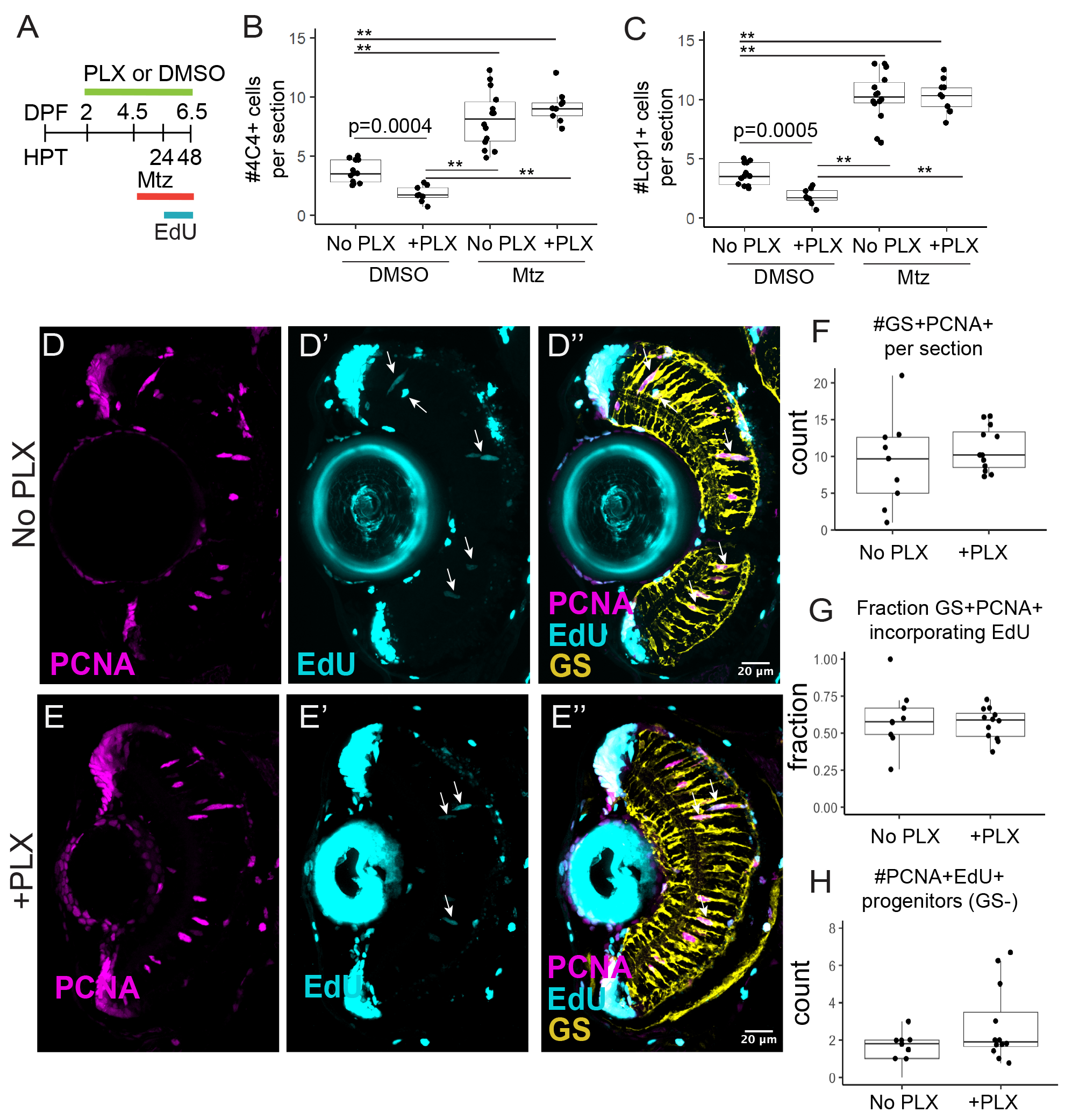

Supplement: S1 Fig — A. Timeline showing treatment of gnat2:nfsb-mCherry;irf8 + /- or gnat2:nfsb-mCherry;irf8-/- fish with Mtz and exposure to PLX3397 (PLX) and EdU. B. Quantification of the number of 4C4 + cells in retinal cryosections of fish at 48 hpt following Mtz treatment, and with (+PLX) or without (No PLX) PLX3397 exposure. C. Quantification of the number of Lcp1 + cells in retinal cryosections of fish at 48 hpt following Mtz treatment, and with (+PLX) or without (No PLX) PLX3397 exposure. D-D.” Images of retinal cryosections from non-PLX treated fish collected at 48 hpt following Mtz treatment, showing PCNA, EdU, and GS signal. E-E.” Images of retinal cryosections from PLX3397 treated fish collected at 48 hpt following Mtz treatment, showing PCNA, EdU, and GS signal. Arrows show GS+PCNA+EdU+ cells in the INL. F. Quantification of the number of GS+PCNA+ cells per retinal section for samples without (No PLX) or with PLX3397 (+PLX) treatment. G. The fraction of GS+PCNA+ cells that were also EdU+ for samples without (No PLX) or with PLX3397 (+PLX) treatment. H. The number of GS-negative, PCNA+EdU+ cells (considered cycling MG-derived progenitors) for samples without (No PLX) or with PLX3397 (+PLX) treatment. ** indicates p < 0.05 (Welch’s test) for comparisons shown in B and C. Differences were not statistically significant for comparisons shown in F, G, and H (Welch’s test). (TIF) [file pone.0344737.s001.tif]

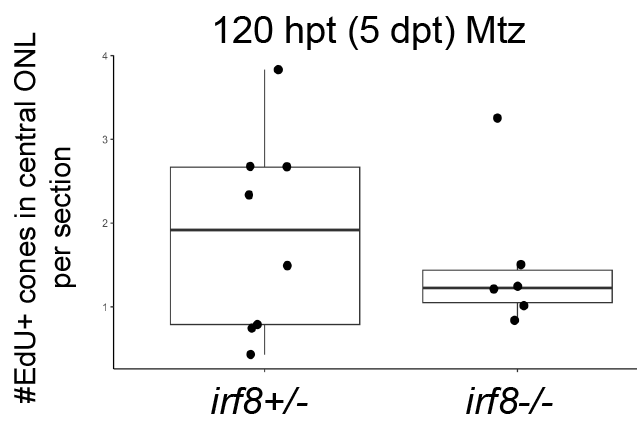

Supplement: S2 Fig — gnat2:nfsb-mCherry;irf8 + /- or gnat2:nfsb-mCherry;irf8-/- larvae were treated with Mtz for 48 hours with EdU immersion following the regime shown in Figure 7E, with EdU washout at 72 hours post treatment (hpt). Retinal cryosections were collected at 120 hpt (5 days post treatment, dpt) and stained for EdU; mCherry was used to identify cones. The graph shows the number of EdU + mCherry+ cells (cones) in retinal cryosections from each genotype detected at 120 hpt (5 dpt). (TIF) [file pone.0344737.s002.tif]

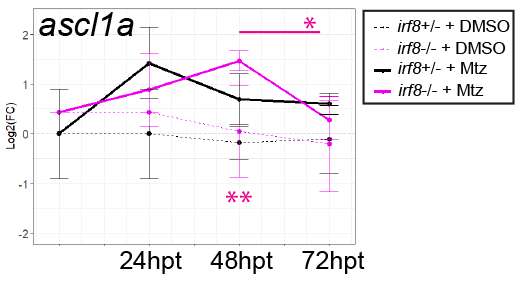

Supplement: S3 Fig — RT-qPCR was performed using RNA collected at the indicated timepoints from pairs of whole larval eyes from gnat2:nfsb-mCherry larvae of either irf8 + /- or irf8-/- genotypes, following DMSO or Mtz treatment. Gene expression for ascl1a was analyzed compared to undamaged (DMSO treated), irf8 + /- samples at 24 hpt, using the 2^ddCt method and graphing as Log2(FC). Complete results from statistical analysis are shown for this data in S1 File. **p = 0.0125 irf8-/- + Mtz 48 hpt vs irf8-/- + DMSO 48 hpt, *p = 0.043; irf8-/- + Mtz 48 hpt vs irf8-/- + Mtz 72 hpt. (TIF) [file pone.0344737.s003.tif]

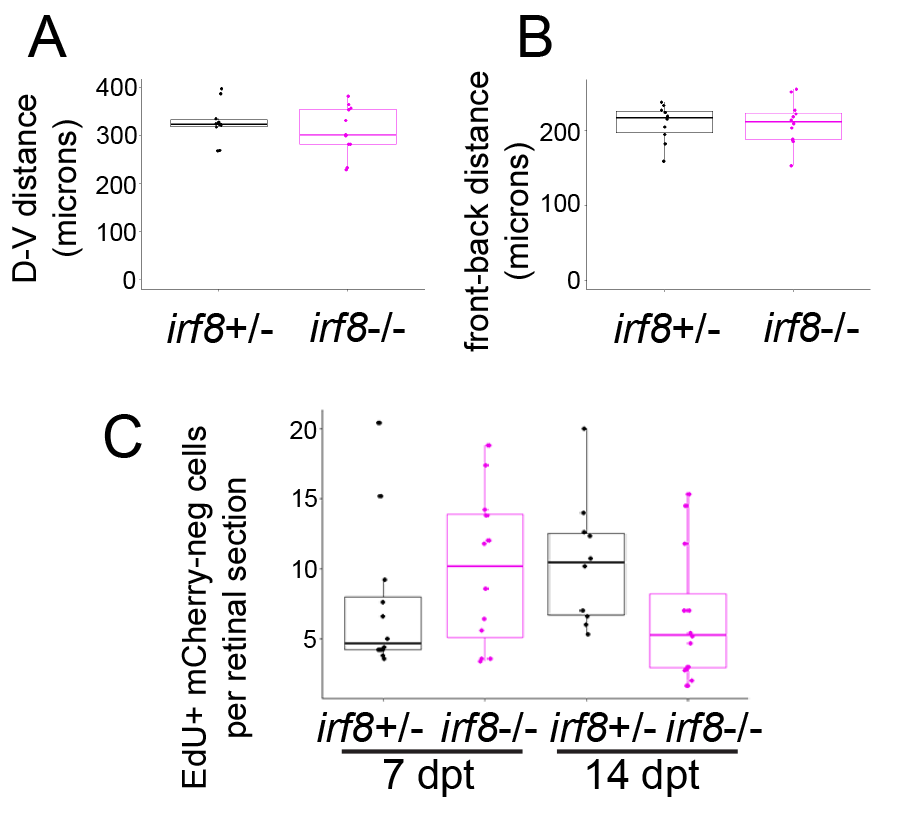

Supplement: S4 Fig — A, B. Eye size measurements between gnat2:nfsb-mCherry;irf8 + /- or gnat2:nfsb-mCherry;irf8-/- larvae at 14 dpt following Mtz treatment for (A) dorsal-ventral (D-V) axis direction diameter length and (B) distance from the lens to the back of the eye boundary (front-back). Measurements were not different between genotypes (Mann-Whitney U-test). C. Counts of EdU+ cells that were mCherry-negative in retinal cryosections for the indicated timepoints and genotypes. Differences between genotypes and between timepoints were not statistically significant (Mann-Whitney U-test for pairwise comparisons between irf8 + /- and irf8-/- at 7 dpt and at 14 dpt, and pairwise comparisons within the same genotype between 7 dpt and 14 dpt). (TIF) [file pone.0344737.s004.tif]

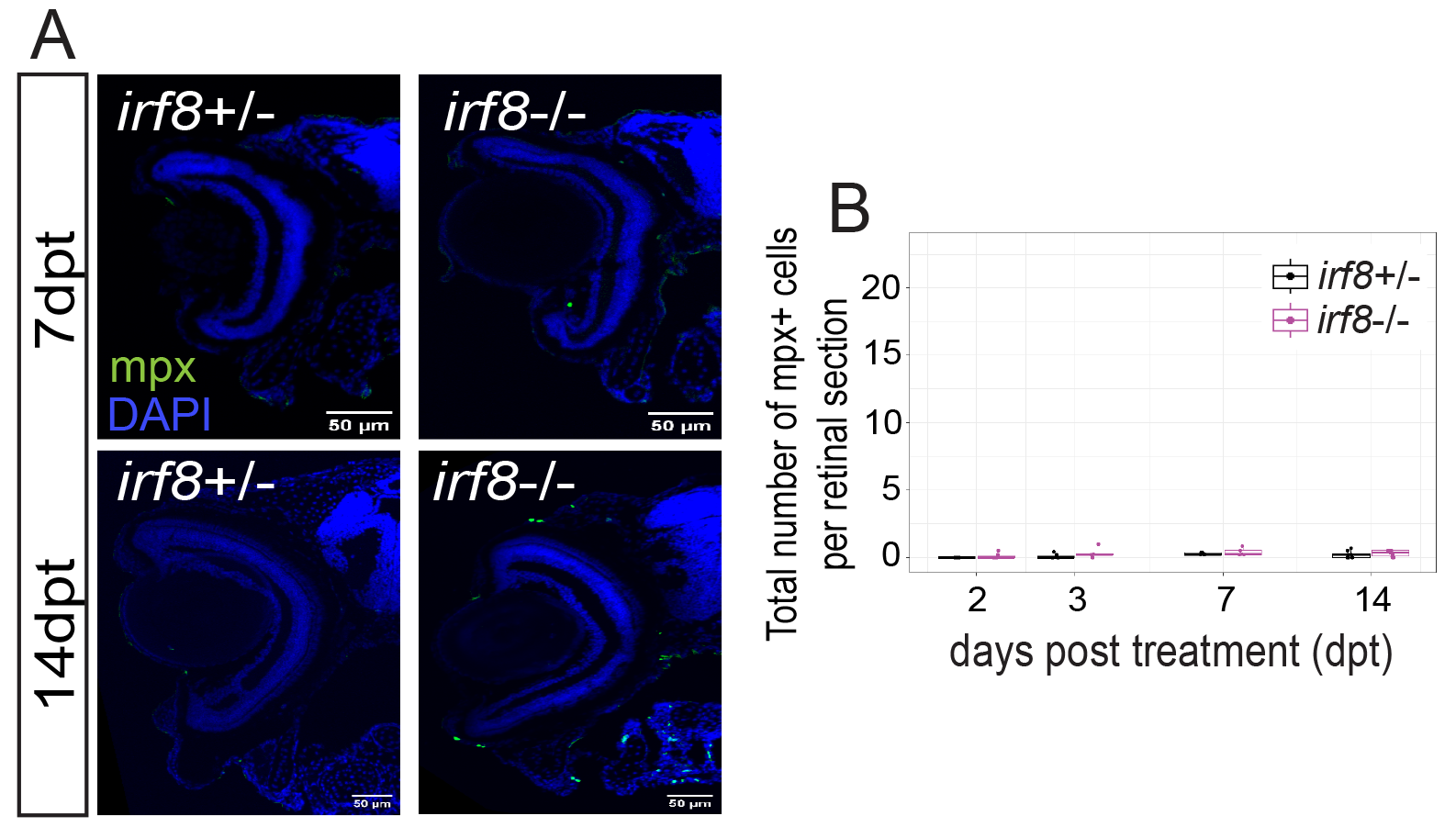

Supplement: S5 Fig — A. We performed staining of retinal cryosections with antibody to Mpx (myeloperoxidase) and DAPI to determine if neutrophils were detected in the responding immune cell populations following cone ablation in gnat2:nfsb-mCherry;irf8 + /- or gnat2:nfsb-mCherry;irf8-/- larvae. Images shown are from samples collected at 7 dpt and at 14 dpt, from both irf8 genotypes as indicated. B. Quantification of the number of Mpx+ cells detected in retinal tissue from the two irf8 genotypes at the indicated timepoints following cone ablation. Mpx+ cells were not abundantly detected in retinas of either genotype, though staining was confirmed by the presence of Mpx+ cells (green) in other tissues outside of retina. 2-way ANOVA (no statistical significance was found). (TIF) [file pone.0344737.s005.tif]
